# The effects of aquatic exercise therapy on blood pressure in elderly people with hypertension: a systematic review and meta-analysis

**DOI:** 10.3389/fcvm.2026.1951070

**Published:** 2026-10-08

**Authors:** Xiaofeng Cheng, Jinsong Luo, Xianhui Liao

**Affiliations:** 1 Department of Infectious Diseases, Shangyu People’s Hospital of Shaoxing, Shaoxing University, Shaoxing, China; 2 Wuchang University of Technology, Wuhan, Hubei, China; 3 Department of Physiotherapy, School of Primary and Allied Health Care, Monash University, Melbourne, VIC, Australia

**Keywords:** aquatic exercise therapy, blood pressure, elderly, heart rate, hypertension, meta-analysis, systematic review

## Abstract

**Background:**

Hypertension remains one of the most common controllable risk factors for cardiovascular disease morbidity and mortality in the elderly. Aquatic exercise therapy (AET), as a safe and low-impact form of exercise, has attracted considerable attention due to its potential cardiovascular benefits; however, its impact on blood pressure control in elderly patients with hypertension remains unclear. This systematic review and meta-analysis aimed to investigate the effects of AET on blood pressure and heart rate in elderly patients with hypertension.

**Methods:**

A comprehensive search of PubMed, Embase, Web of Science, the Cochrane Library, and CINAHL was conducted from inception to July 2026. Randomized controlled trials (RCTs) evaluating the effects of AET compared with control interventions in older adults with hypertension were included. The primary outcomes were systolic blood pressure (SBP) and diastolic blood pressure (DBP), while HR was considered a secondary outcome. Random-effects models were applied for meta-analysis, and subgroup analyses were performed based on the type of control intervention.

**Results:**

Four RCTs involving 123 participants were included in this meta-analysis. Compared with control conditions, aquatic exercise therapy (AET) significantly reduced systolic blood pressure (SBP) (MD −10.98 mmHg, 95% CI −17.41 to −4.56, *P* = 0.001; I² = 29%), diastolic blood pressure (DBP) (MD −5.97 mmHg, 95% CI −6.57 to −5.36, *P* = 0.001; I² = 0%), and heart rate (HR) (MD −5.91 beats/min, 95% CI −6.70 to −5.12, *P* = 0.001; I² = 0%). According to the GRADE assessment, the certainty of evidence for the main outcomes was rated as moderate.

**Conclusions:**

AET may be an effective non-pharmacological intervention for improving blood pressure control and reducing heart rate in older adults with hypertension. However, further well-designed RCTs with larger sample sizes are needed to confirm its long-term clinical effects.

**Systematic Review Registration:**

https://www.crd.york.ac.uk/PROSPERO/view/CRD420261462262, PROSPERO: CRD420261462262.

## 1 Introduction

Hypertension is one of the most common chronic diseases worldwide and a major controllable risk factor for cardiovascular disease morbidity and mortality (1). According to the World Health Organization (WHO), approximately 1.28 billion adults aged 30 to 79 worldwide suffer from hypertension, and the prevalence increases significantly with age (2). Older adults are particularly susceptible to hypertension due to age-related vascular changes, including arteriosclerosis, endothelial dysfunction, and impaired autonomic regulation (3). These pathophysiological changes lead to persistently elevated blood pressure and increase the risk of cardiovascular and renal complications, including stroke, myocardial infarction, heart failure, chronic kidney disease, and premature death (1, 4). Therefore, effective blood pressure control is a crucial public health priority in aging societies (1, 2).

Lifestyle modification is regarded as a cornerstone of hypertension management and is strongly recommended by contemporary international guidelines (5, 6). Among non-pharmacological interventions, regular physical activity has consistently been shown to lower blood pressure and improve cardiovascular health and is recommended by the European Society of Cardiology (4), the European Society of Hypertension (ESH), and the American College of Cardiology/American Heart Association (ACC/AHA) (5, 7). Despite these well-established benefits, participation in conventional land-based exercise remains challenging for many older adults (8). In older adults with hypertension, long-term engagement in exercise may be further affected by reduced exercise tolerance, fatigue, concerns about exercise-related cardiovascular symptoms or blood pressure fluctuations, polypharmacy, and the presence of multiple chronic conditions. Age-related conditions, including osteoarthritis, obesity, impaired balance, frailty, and fear of falling, may limit engagement in weight-bearing exercise, resulting in difficulty initiating or maintaining conventional exercise programmes and potentially reducing the long-term benefits of lifestyle-based blood pressure management (8, 9).

Aquatic exercise therapy (AET) has emerged as a promising alternative to land-based exercise because it provides a safe and comfortable environment for physical activity while minimizing mechanical stress on the musculoskeletal system (10). The therapeutic properties of water, including buoyancy, hydrostatic pressure, viscosity, and thermal effects, promote athletic performance by reducing joint load, while providing multidirectional resistance and stimulating the cardiovascular system (10, 11). Furthermore, hydrostatic pressure enhances venous return and cardiac preload, thereby increasing stroke volume and cardiovascular efficiency; while warm water immersion has been shown to reduce sympathetic nervous system activity and decrease peripheral vascular resistance (11). These physiological adaptations provide a plausible mechanistic basis for the hypotensive effects of AET and suggest its potential to improve cardiovascular function and exercise tolerance in elderly patients with hypertension (10, 12).

Although an increasing number of randomized controlled trials (RCTs) have investigated the effects of AET on blood pressure in older adults with hypertension, the available evidence remains inconclusive (13). Considerable heterogeneity in intervention protocols, comparator interventions, treatment duration, and methodological quality has limited the comparability of individual studies and hindered definitive conclusions regarding the effectiveness of AET (7, 13). Furthermore, previous systematic reviews have primarily examined the effects of aquatic exercise in broader populations or on general cardiovascular and functional outcomes rather than specifically focusing on blood pressure control in older adults with hypertension (14, 15). Consequently, the overall efficacy of AET for this population has yet to be comprehensively established.

Therefore, this systematic review and meta-analysis aim to synthesize existing evidence from randomized controlled trials to evaluate the effects of aquatic exercise therapy on systolic blood pressure, diastolic blood pressure, and heart rate in elderly patients with hypertension. By quantitatively integrating existing evidence, this study aims to elucidate the clinical efficacy of aquatic exercise therapy and provide evidence-based support for exercise prescriptions and non-pharmacological treatment strategies for hypertension in the elderly.

## 2 Methods

The conduct and reporting of this systematic review and meta-analysis follow the Preferred Reporting Items for Systematic Reviews and Meta-analyses (PRISMA) 2020 statement (16). This evaluation protocol has been prospectively registered with the International Registry for Prospective Systems Reviews (PROSPERO) under registration number CRDCRD420261462262.

### 2.1 Eligibility criteria

The eligibility criteria were established according to the Population, Intervention, Comparator, Outcomes, and Study design (PICOS) framework (Supplementary Table 1) (17). Studies were considered eligible if they met the following criteria. (1) Participants: Studies were eligible if they included older adults with hypertension diagnosed according to recognized clinical guidelines or physician diagnosis (6). Participants were generally aged ≥60 years or classified as older adults in the original studies, irrespective of sex, ethnicity, disease duration, or antihypertensive medication use; (2) Intervention: Eligible studies investigated structured aquatic exercise interventions performed in a water environment. Interventions included water-based aerobic exercise, aquatic resistance exercise, aquatic combined exercise, and aquatic exercise therapy. The included interventions were required to involve active, supervised, or prescribed physical exercise conducted in water. Swimming was considered eligible only when it was performed as a structured exercise intervention rather than recreational activity. No restrictions were placed on exercise intensity, session duration, frequency, or intervention period. Studies in which aquatic exercise was combined with other interventions were eligible only if the co-interventions were applied equally to both the intervention and comparator groups.; (3) Control group: Eligible comparator interventions included conventional rehabilitation, land-based exercise, usual care, no intervention, or other non-aquatic control interventions; (4) Outcome measures: The primary outcomes were systolic blood pressure (SBP) and diastolic blood pressure (DBP). Secondary outcomes included mean arterial pressure (MAP), resting heart rate, cardiorespiratory fitness, vascular function, body composition, health-related quality of life, and adverse events. Studies were required to report sufficient quantitative data for quantitative synthesis. (5) Study design: randomized controlled trial (RCT).

### 2.2 Information sources

A comprehensive literature search was conducted in PubMed, Embase, Web of Science, the Cochrane Library, and CINAHL from database inception to July 17, 2026. The search strategy was developed in accordance with the recommendations of the Preferred Reporting Items for Systematic Reviews and Meta-Analyses (PRISMA) 2020 Statement and combined controlled vocabulary [e.g., Medical Subject Headings (MeSH) in PubMed] with free-text terms related to aquatic exercise therapy, hypertension, and older adults (6). The detailed search strategy for PubMed is presented in Supplementary Table 2, and equivalent search strategies were adapted for the other electronic databases. To ensure completeness of the search, the reference lists of all included studies and relevant systematic reviews were manually screened to identify additional eligible studies. Only studies published in English and involving human participants were considered eligible.

### 2.3 Search strategy

The search strategy was developed according to the PICOS framework and independently performed by two reviewers (Xianhui Liao and Jinsong Luo). Controlled vocabulary terms [i.e., Medical Subject Headings (MeSH) in PubMed and Emtree terms in Embase] were combined with free-text keywords to maximize search sensitivity (17). The search strategy included three core concepts: (1) aquatic exercise therapy, (2) hypertension, and (3) older adults.

Search terms related to aquatic exercise therapy included Aquatic Therapy, aquatic therapy, aquatic exercise, water-based exercise, water exercise, hydrotherapy, aquatic training, pool exercise, aquatic rehabilitation, water-based rehabilitation, water walking, aqua jogging, aquatic resistance exercise, aquatic balance training, and swimming. Terms related to hypertension include Hypertension, hypertension, hypertensive, and high blood pressure. Terms describing the target population included Aged, Aged, elderly, older adults, older people, ageing, aging, geriatric, and senior. Appropriate Boolean operators (AND and OR) were used to combine the search terms.

The search strategy was initially developed for PubMed and subsequently adapted for Embase, Web of Science, the Cochrane Library, and CINAHL according to the indexing system of each database. No restrictions were imposed on publication year. The complete electronic search strategies for all databases are provided in Supplementary Table 2.

To ensure the comprehensiveness of the literature search, the reference lists of all included studies and relevant systematic reviews were manually screened to identify additional eligible studies (17).

### 2.4 Study selection

All records identified through the electronic database search were imported into Covidence for the removal of duplicate records. Subsequently, two reviewers (Xianhui Liao and Jinsong Luo) independently screened the titles and abstracts of all retrieved studies according to the predefined eligibility criteria. Full-text articles were then obtained and independently assessed for eligibility by the same two reviewers.

Studies were excluded during the full-text screening stage if they did not meet the predefined inclusion criteria with respect to the study population, intervention, comparator, outcomes, or study design. The reasons for exclusion at the full-text review stage were documented. The overall selection process is presented in the Preferred Reporting Items for Systematic Reviews and Meta-Analyses (PRISMA) 2020 flow diagram (Figure 1).

**Figure 1:**
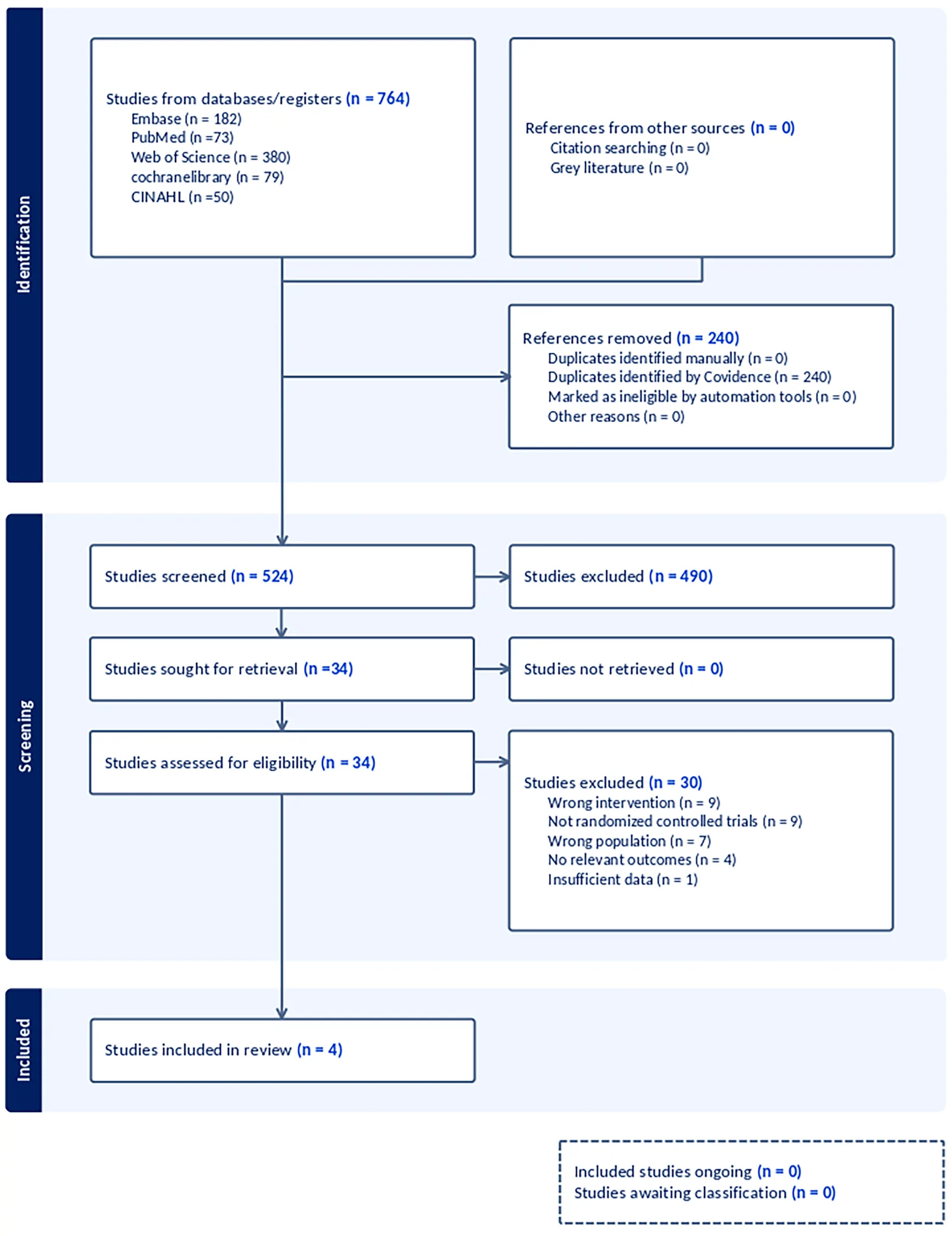
Flowchart of literature screening and inclusion for systematic review and meta-analysis.

Any disagreements between the two reviewers were resolved through discussion. If consensus could not be reached, a third reviewer (Xiaofeng Cheng) was consulted to make the final decision.

### 2.5 Data collection process and data extraction

Two reviewers (Xianhui Liao and Jinsong Luo) independently extracted data from all included studies using a standardized data extraction form developed before the review. The extracted data was cross-checked by both reviewers to ensure accuracy and completeness. Any discrepancies were resolved through discussion, and a third reviewer (Xiaofeng Cheng) was consulted when consensus could not be reached (17).

The following information was extracted from each included study: (1) study characteristics, including first author, year of publication, country and study design; (2) participant characteristics, including sample size, age, sex, diagnostic criteria for hypertension and baseline characteristics; (3) intervention characteristics, including type of aquatic exercise therapy, exercise intensity, exercise frequency, duration of treatment, details of intervention and control interventions; and (4) outcome measures, including systolic blood pressure (SBP), diastolic blood pressure (DBP), mean arterial pressure (MAP), resting heart rate (HR), cardiopulmonary function, vascular function, body composition, health-related quality of life and adverse events.

For continuous outcome measures, we extracted the mean, standard deviation (SD), and sample size for both the intervention and control groups whenever possible. When outcome data were reported in other formats, we converted the relevant statistics to a standardized format according to the recommendations of the Cochrane Manual of Interventions for Systematic Reviews to facilitate quantitative synthesis analysis. If key data was missing or unclear, we contacted the relevant authors via email for more information.

### 2.6 Quality assessment

The methodological quality of the included randomized controlled trials (RCTs) was independently assessed by two reviewers (Xianhui Liao and Jinsong Luo) using the Cochrane Risk of Bias 2 (RoB 2) tool for randomized trials, as recommended in the Cochrane Handbook for Systematic Reviews of Interventions (18). Any disagreements were resolved through discussion, and a third reviewer (Xiaofeng Cheng) was consulted when consensus could not be reached.

The risk of bias was assessed across five domains: (1) bias arising from the randomization process; (2) bias due to deviations from intended interventions; (3) bias due to missing outcome data; (4) bias in measurement of the outcome; and (5) bias in selection of the reported result. Each domain was judged as having a “low risk of bias,” “some concerns,” or a “high risk of bias” according to the signaling questions and assessment criteria specified in the RoB 2 tool. An overall risk-of-bias judgment was then assigned to each study based on the domain-level assessments.

To ensure the accuracy and consistency of the risk-of-bias assessment, the extracted study characteristics and outcome data were independently cross-checked by the two reviewers against the original publications. Any discrepancies in the extracted data or risk-of-bias judgments were resolved through discussion and, when necessary, consultation with a third reviewer. If key data was missing or unclear, we contacted the relevant authors via email for more information.

### 2.7 Data synthesis

All statistical analyses were performed using Review Manager (RevMan) version 5.4 (The Cochrane Collaboration, Copenhagen, Denmark) (19). Continuous outcomes were summarized as mean differences (MDs) with corresponding 95% confidence intervals (CIs) because all included studies reported outcomes using consistent measurement units. A random-effects model was applied to account for potential clinical and methodological heterogeneity among the included studies (17).

Statistical heterogeneity was assessed using Cochran’s *Q* test and quantified with the I² statistic. According to the recommendations of the Cochrane Handbook for Systematic Reviews of Interventions (17, 20). I² values of approximately 25%, 50%, and 75% were considered to represent low, moderate, and high heterogeneity, respectively. Statistical significance was defined as a two-sided *P* value < 0.05.

Meta-analyses were performed for the primary outcomes, including systolic blood pressure (SBP) and diastolic blood pressure (DBP), and the secondary outcome, resting heart rate (HR).

To investigate potential sources of heterogeneity, prespecified subgroup analyses were conducted according to the type of comparator intervention, including non-exercise control and land-based exercise. Differences between subgroups were evaluated using the test for subgroup differences implemented in RevMan (37).

For outcome measures with a sufficient number of included studies, we used funnel plots for a visual assessment of publication bias. Because each outcome measure had fewer than 10 studies, we interpreted the funnel plot results with caution, following the recommendations of the Cochrane Manual of Interventions for Systematic Reviews.

### 2.8 Certainty of evidence assessment

The certainty of evidence for each outcome was independently assessed by two reviewers (Xianhui Liao and Jinsong Luo) using the Grading of Recommendations Assessment, Development and Evaluation approach (21). The certainty of evidence was evaluated across five domains: risk of bias, inconsistency, indirectness, imprecision, and publication bias, in accordance with the GRADE Working Group recommendations. The overall certainty of evidence for each outcome was rated as high, moderate, low, or very low.

Because all included studies were randomized controlled trials, the certainty of evidence was initially considered high and was downgraded when concerns were identified in one or more GRADE domains. Any disagreements between the two reviewers were resolved through discussion, and a third reviewer (Xiaofeng Cheng) was consulted when consensus could not be reached.

A Summary of Findings (SoF) table was generated using the GRADEpro Guideline Development Tool (GRADEpro GDT) to summarize the certainty of evidence for the primary and secondary outcomes (22).

## 3 Results

### 3.1 Search process

The literature search identified a total of 764 records through electronic database searching, including PubMed (*n* = 73), Embase (*n* = 182), Web of Science (*n* = 380), the Cochrane Library (*n* = 79), and CINAHL (*n* = 50). No additional records were identified through citation searching or grey literature sources. After the removal of 240 duplicate records using Covidence, 524 studies remained for title and abstract screening. After title and abstract screening, 490 articles were excluded because they did not meet the pre-defined inclusion criteria. The remaining 34 full-text articles were retrieved and eligibility assessed. Among them, 30 studies were excluded for the following reasons: inappropriate intervention (*n* = 9), non-randomized controlled trials (*n* = 9), inappropriate study population (*n* = 7), failure to report relevant outcome measures (*n* = 4), and insufficient data for quantitative meta-analysis (*n* = 1). Ultimately, four randomized controlled trials (RCTs) involving 123 participants met the eligibility criteria and were included in the qualitative and quantitative syntheses (23–28). The study selection process is illustrated in the PRISMA 2020 flow diagram (Figure 1).

### 3.2 Description of participants

This meta-analysis included four RCTs involving 123 participants. The mean age of the participants was 68.2 years. Of all participants, 20 were male (16.26%) and 103 were female (83.73%), indicating a predominantly female population. All four included studies recruited older adults with hypertension. However, detailed hypertension categories or severity classifications were not consistently reported across the included studies. The characteristics of the included studies are summarized in Table 1. All included studies recruited older adults with hypertension. However, the definition and classification of hypertension varied among studies. Júnior et al. (2020) included older women with hypertension who were receiving regular treatment for blood pressure control, whereas Kim et al. (2016) recruited older women with blood pressure levels exceeding 140/90 mmHg at screening. Ruangthai et al. (2020) included older adults with hypertension who continued their usual medication throughout the intervention period. Sosner et al. (2019) included participants with office systolic blood pressure ≥130 mmHg and/or diastolic blood pressure ≥85 mmHg who were receiving antihypertensive treatment. Detailed participant characteristics, hypertension-related information, and medication status are summarized in Table 2.

**Table 1:** General participant and study characteristics.

Characteristics	Number (%)	Mean
Total sample size	123 (100.0)	
Age, years	68.2	
Sex		
Male participants, *n* (%)	20 (16.26)	
Female participants, *n* (%)	103 (83.73)	
Country		
Korea	1 (25.00)	
Brazil	1 (25.00)	
Thailand	1 (25.00)	
Canada	1 (25.00)	
Language		
English	4 (100.0)	

**Table 2:** Characteristics of the included studies.

First Author (Year, Language, Location)	Sample characteristics						
	T/C	Sample size	Sex	Age	Intervention/Control	Hypertension characteristics	Antihypertensive medication status
Júnior et al. (2020, English, Brazil)	T	10	Male/ female: 0/10	70 ± 2	swim	Older women (>60 years) with hypertension; participants were receiving regular treatment for blood pressure control.	All participants were receiving regular treatment for blood pressure control.
	C	10	Male/ female: 0/10	65 ± 3	Land group		
Kim et al. (2016, English, Korea)	T	25	Male/ female: 0/25	73.22 ± 3.30	swim	Older women with blood pressure >140/90 mmHg at screening.	NR
	C	25	Male/ female: 0/25	72.65 ± 4.28	control		
Ruangthai et al. (2020, English, Thailand)	T	13	Male/ female: 1/12	69.7 ± 6.5	swim	Older adults with hypertension	Continued usual medication
	C	12	Male/ female: 5/7	66.7 ± 5.8	control		
Sosner et al. (2019, English, Canada)	T	14	Male/ female: 5/9	63 ± 9	swim	Participants with office SBP ≥130 mmHg and/or DBP ≥85 mmHg	23/42 (55%) participants were receiving antihypertensive treatment.
	C	14	Male/ female: 9/5	65 ± 8	Land group		

### 3.3 Description of the interventions and comparators

All included studies investigated structured aquatic exercise interventions among older adults with hypertension. However, intervention protocols varied in terms of duration, frequency, session duration, exercise intensity, and aquatic environment. Júnior et al. (2020) (23) implemented a 12-week aquatic exercise program consisting of five sessions per week, with each session lasting 60 min. The comparator group participated in a land-based exercise program with the same intervention duration. Kim et al. (2016) (27) investigated a 12-week aquatic exercise program performed three times per week, with each session lasting 50 min, whereas the control group received no structured exercise intervention. Ruangthai et al. (2020) (24) applied a 12-week water-based combined exercise program consisting of three 60-minute sessions per week. The intervention was performed in water maintained at 26–29 °C, with immersion between the xiphoid process and shoulders. Sosner et al. (2019) (25) conducted a 2-week aquatic high-intensity interval training program using an immersed ergocycle. Participants completed three 30-minute sessions per week in water maintained at 30 °C, with immersion up to chest level. The detailed dosage, intensity, and aquatic environmental parameters of each intervention protocol are presented in Table 3.

**Table 3:** Dosage of intervening program.

First Author (Year, Language, Location)	T/C	Dosage				Water temperature	Immersion level
		Intervention/Control	Duration (weeks)	Frequency (per week)	Intensity (min)		
Júnior et al. (2020, English, Brazil)	T	Aquatic exercise therapy	12	5	60	NR	NR
	C	Land group	12	5	60		
Kim et al. (2016, English, Korea)	T	Aquatic exercise therapy (aquarobics)	12	3	50	NR	NR
	C	control	/	/	/		
Ruangthai et al. (2020, English, Thailand)	T	Water-based	12	3	60	26–29 °C	Between xiphoid process and shoulders
	C	control	/	/	/		
Sosner et al. (2019, English, Canada)	T	Aquatic high-intensity interval training	2	3	30	30 °C	Up to chest level
	C	Land group	2	3	30		

### 3.4 Outcome measures

The outcome measures reported across the included RCTs varied among studies. Júnior et al. (2020) (23) assessed HR, SBP, and DBP. Kim et al. (2016) (27) reported SBP, DBP, mean blood pressure, and HR. Ruangthai et al. (2020) (24) evaluated FBG, TC, LDL-C, HDL-C, non-HDL cholesterol, TC/HDL-C ratio, AI, AIP, and LCI. Sosner et al. (2019) (25) assessed SBP, DBP, and HR. Overall, SBP and DBP were the most frequently reported outcomes, while HR was also assessed in several studies. The detailed outcome measures reported by each included study are presented in Table 4.

**Table 4:** Outcome indicators, adverse events, and follow-ups included in the studies.

First Author (Year, Language, Location)	T/C	Outcome	adverse events (Yes/ No/No report)	follow-up (Yes/ No/No report)
Júnior et al. (2020, English, Brazil)	T	HR, SBP, DBP	No report	No report
	C			
Kim et al. (2016, English, Korea)	T	SBP, DBP, MBP, HR	No report	No report
	C			
Ruangthai et al. (2020, English, Thailand)	T	FBG, TC, LDL-C, HDL-C,non-HDL-C, TC/HDL-C, LDL-C/HDL-C, AI, AIP, LCI	Yes (*n* = 4): 2 reported personal con- straints (drop-out), 2 reported illness (com-pleted the exercise though didnot perform post-test)	Yes
	C			
Sosner et al. (2019, English, Canada)	T	SBP, DBP, HR	No report	No report
	C			
	C			

HR, heart rate; SBP, systolic blood pressure; DBP, diastolic blood pressure; MBP, mean blood pressure; FBG, fasting blood glucose; TC, total cholesterol; LDL-C, low-density lipoprotein cholesterol; HDL-C, high-density lipoprotein cholesterol; non-HDL-C, non-high-density lipoprotein cholesterol; TC/HDL-C, total cholesterol to high-density lipoprotein cholesterol ratio; LDL-C/HDL-C, low-density lipoprotein cholesterol to high-density lipoprotein cholesterol ratio; AI, atherogenic index; AIP, atherogenic index of plasma; LCI, lipid accumulation product index.

### 3.5 Adherence, retention, and adverse events

Reporting of participant adherence and retention varied across the included studies. None of the included RCTs reported quantitative measures of exercise adherence, such as attendance or compliance rates. Only one study included a follow-up assessment after completion of the intervention, whereas no follow-up information was reported in the remaining studies.

Adverse events were infrequently reported. Among the four included studies, only Ruangthai et al. (2020) documented adverse events. A total of four adverse events were reported, including two participants who withdrew because of personal complaints and two participants who developed illness and completed the intervention but did not undergo the post-intervention assessment. No other included studies reported adverse events (Table 4).

### 3.6 Risk of bias

The risk of bias of the four included randomized controlled trials was assessed using the Cochrane Risk of Bias 2 (RoB 2) tool for randomized trials, and the results are presented in Figure 2 and Supplementary Table 3. Overall, all four included studies were judged to have some concerns regarding the overall risk of bias. No study was judged to have a low or high overall risk of bias.

**Figure 2:**
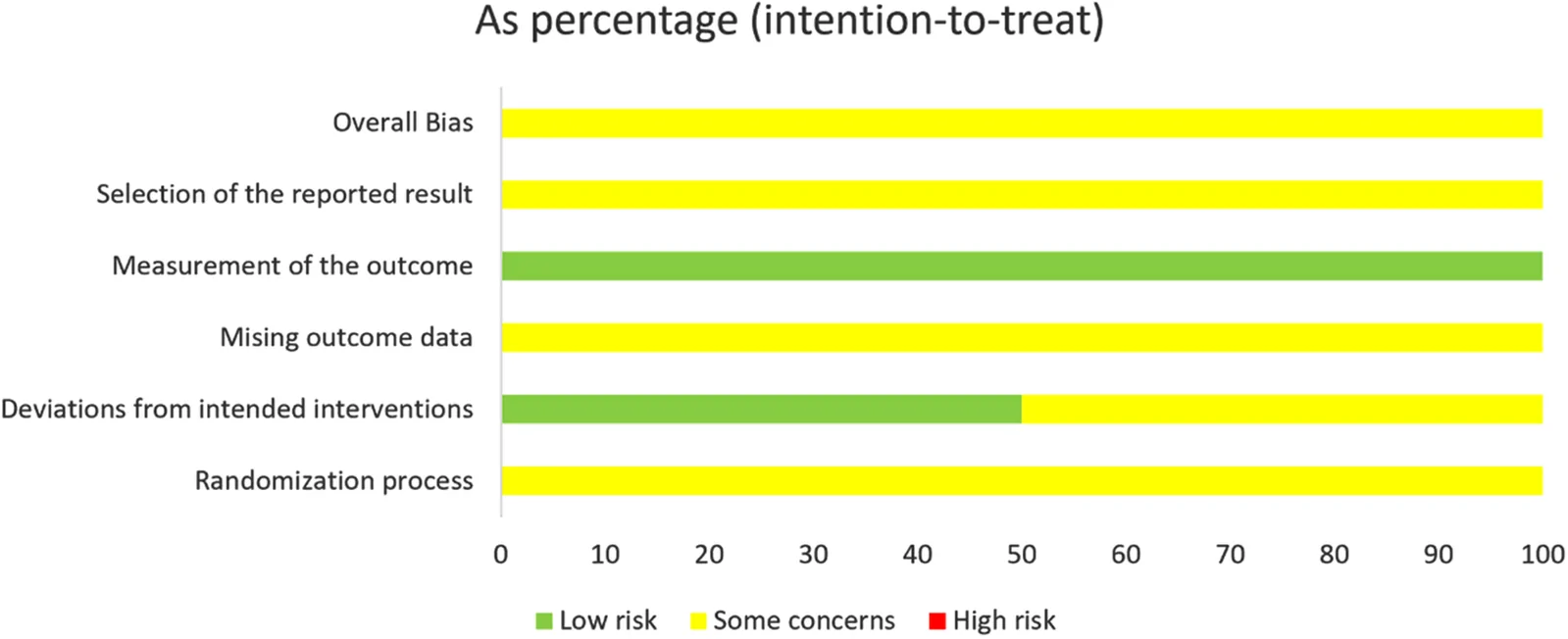
Risk of bias graph.

Regarding bias arising from the randomization process (Domain 1), all four studies were judged to have some concerns. Although the studies reported that participants were randomly assigned to the intervention and comparator groups, insufficient details were provided regarding the specific methods used to generate the random allocation sequence and/or the procedures used to ensure adequate allocation. Therefore, the randomization process could not be considered at low risk of bias.

For bias due to deviations from intended interventions (Domain 2), two studies were judged to have a low risk of bias, whereas two studies were judged to have some concerns. The lack of blinding of participants and personnel was considered in the context of the nature of aquatic exercise interventions, for which participant and intervention-provider blinding is generally difficult. However, the absence of blinding alone was not considered sufficient to indicate a high risk of bias. The studies judged to have some concerns did not provide sufficient information to determine whether deviations from the intended interventions may have affected the estimated intervention effects.

Regarding bias due to missing outcome data (Domain 3), all four studies were judged to have some concerns. Information regarding participant attrition, exclusions, and the handling of missing outcome data was not sufficiently detailed to confidently determine whether missingness could have influenced the estimated intervention effects. Therefore, this domain was conservatively judged as having some concerns across all included studies.

For bias in measurement of the outcome (Domain 4), all four studies were judged to have a low risk of bias. The primary outcomes, including systolic blood pressure, diastolic blood pressure, and heart rate, were assessed using objective physiological measurements. These outcomes are relatively unlikely to be influenced by subjective judgment of outcome assessors, and there was no clear indication that outcome measurement was substantially affected by knowledge of the intervention received.

Regarding bias in selection of the reported result (Domain 5), all four studies were judged to have some concerns. Prespecified statistical analysis plans or trial protocols were not sufficiently available or reported, making it difficult to determine whether the reported findings were selected from multiple eligible analyses, outcome measurements, or time points. However, there was insufficient evidence to classify any study as having a high risk of bias in this domain.

Overall, all four included studies were judged to have some concerns regarding the overall risk of bias, primarily because of limitations in the reporting of the randomization process, missing outcome data, and selection of the reported results. Nevertheless, all studies were judged to have a low risk of bias for measurement of the outcomes, and two studies were judged to have a low risk of bias regarding deviations from intended interventions. These findings indicate that, although methodological limitations and reporting uncertainties were present, there was no clear evidence of a high overall risk of bias among the included studies.

### 3.7 Primary outcomes

#### 3.7.1 Systolic blood pressure

Three randomized controlled trials involving 98 participants evaluated the effects of aquatic exercise therapy (AET) on SBP. The pooled analysis demonstrated that AET significantly reduced SBP compared with the control interventions (MD −10.98 mmHg, 95% CI −17.41 to −4.56, *P* = 0.001). There was low heterogeneity among the studies (I² = 29%) (Figure 3 and Supplementary Table 4).

**Figure 3:**
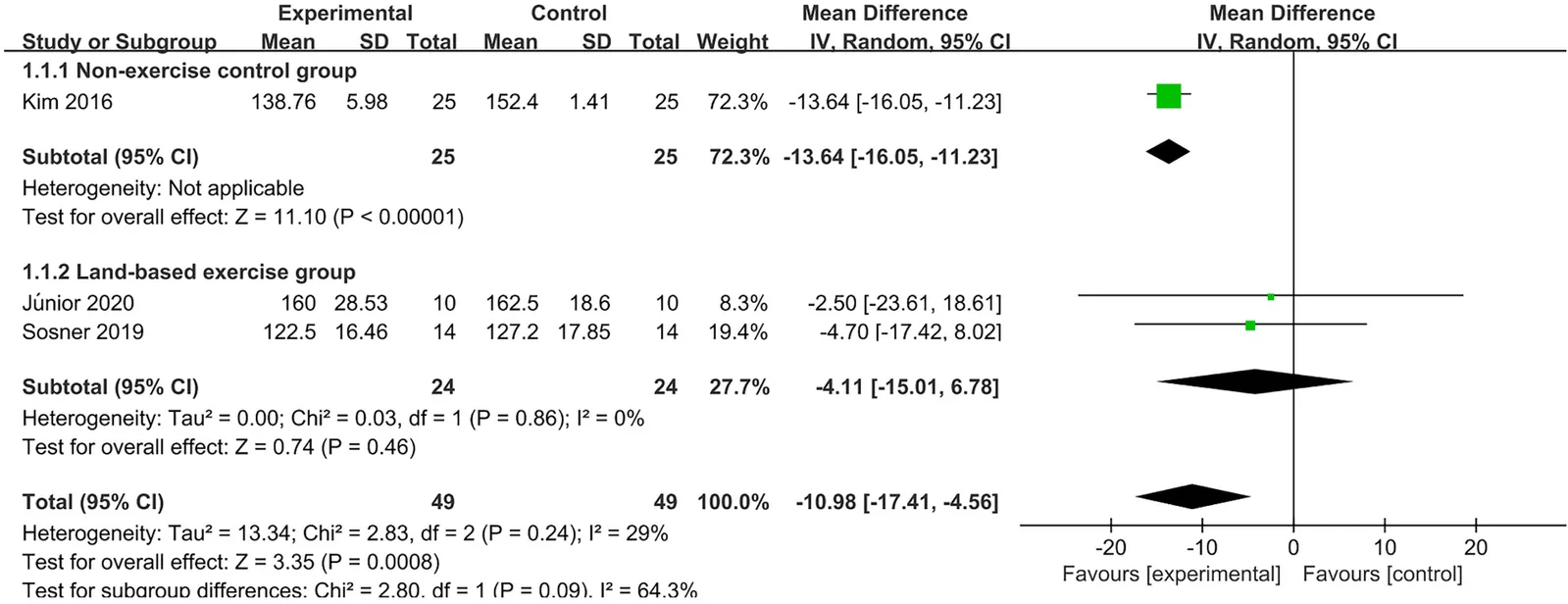
Forest plot showing the effects of aquatic exercise therapy on systolic blood pressure in elderly people with hypertension.

Subgroup analyses were conducted according to comparator type to explore whether the effects of AET differed between non-exercise and land-based exercise comparators. For SBP, AET significantly reduced SBP compared with non-exercise controls (MD −13.64 mmHg, 95% CI −16.05 to −11.23, *P* < 0.00001), whereas the pooled effect compared with land-based exercise was not statistically significant (MD −4.11 mmHg, 95% CI −15.01 to 6.78, *P* = 0.46). The test for subgroup differences showed no statistically significant difference between the two comparator subgroups (*χ*² = 2.80, df = 1, *P* = 0.09; I² = 64.3%) (Supplementary Table 5).

#### 3.7.2 Diastolic blood pressure

Three randomized controlled trials involving 98 participants assessed DBP. Overall, AET significantly reduced DBP compared with the control interventions (MD −5.97 mmHg, 95% CI −6.57 to −5.36, *P* = 0.001). There was no heterogeneity among the tudies (I² = 0%). (Figure 4 and Supplementary Table 4).

**Figure 4:**
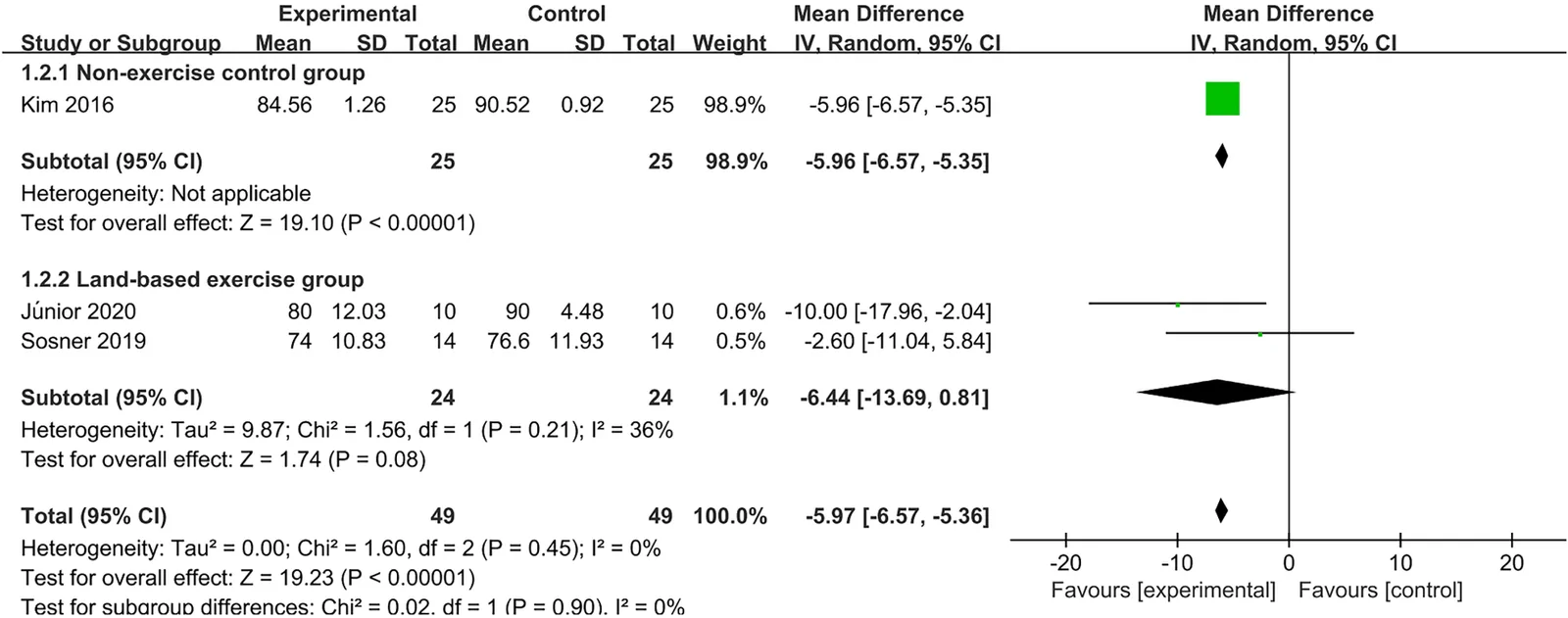
Forest plot showing the effects of aquatic exercise therapy on diastolic blood pressure in elderly people with hypertension.

Subgroup analyses were conducted according to comparator type to explore whether the effects of AET differed between non-exercise and land-based exercise comparators. For DBP, AET significantly reduced DBP compared with non-exercise controls (MD −5.96 mmHg, 95% CI −6.57 to −5.35, *P* < 0.00001). Compared with land-based exercise, the pooled effect was also in favour of AET but did not reach statistical significance (MD −6.44 mmHg, 95% CI −13.69 to 0.81, *P* = 0.08). The test for subgroup differences showed no statistically significant difference between the two comparator subgroups (*χ*² = 0.02, df = 1, *P* = 0.90; I² = 0%) (Supplementary Table 5).

#### 3.7.3 Resting heart rate

Four randomized controlled trials involving 98 participants evaluated resting HR. The pooled analysis showed that AET significantly reduced resting HR compared with the control interventions (MD −5.91 beats/min, 95% CI −6.70 to −5.12, *P* = 0.001). There was no heterogeneity among the studies (I² = 0%) (Figure 5 and Supplementary Table 4).

**Figure 5:**
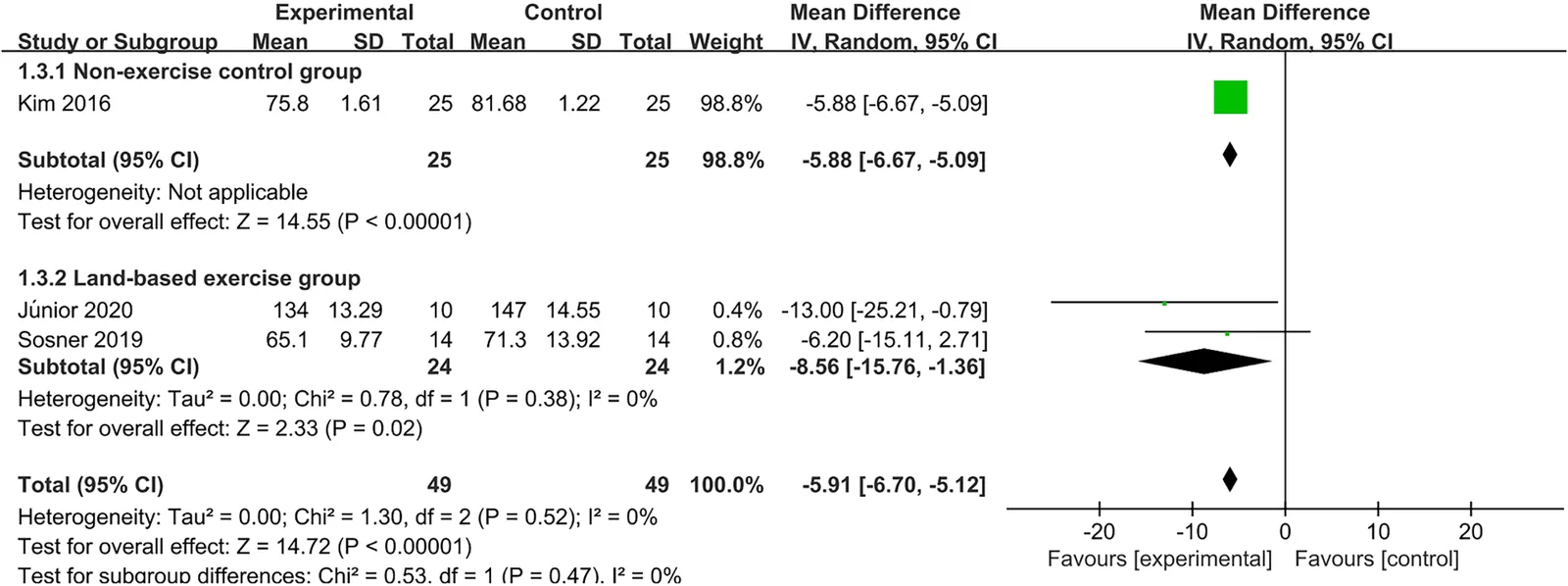
Forest plot showing the effects of aquatic exercise therapy on heart rate in elderly people with hypertension.

Subgroup analyses were conducted according to comparator type to explore whether the effects of AET differed between non-exercise and land-based exercise comparators. For HR, AET significantly reduced HR compared with both non-exercise controls (MD −5.88 beats/min, 95% CI −6.67 to −5.09, *P* < 0.00001) and land-based exercise (MD −8.56 beats/min, 95% CI −15.76 to −1.36, *P* = 0.02). The test for subgroup differences showed no statistically significant difference between the two comparator subgroups (*χ*² = 0.53, df = 1, *P* = 0.47; I² = 0%) (Supplementary Table 5).

### 3.8 Publication bias

We plotted funnel plots for the primary outcome measures (systolic and diastolic blood pressure) and the secondary outcome measure (heart rate), as shown in Figure 6. Visually, the distribution of studies across the three outcome measures appears unbiased. However, since each meta-analysis included fewer than 10 studies, the funnel plots should be interpreted with caution due to their limited ability to detect publication bias. Therefore, no definitive conclusion can be drawn regarding the existence of publication bias.

**Figure 6:**
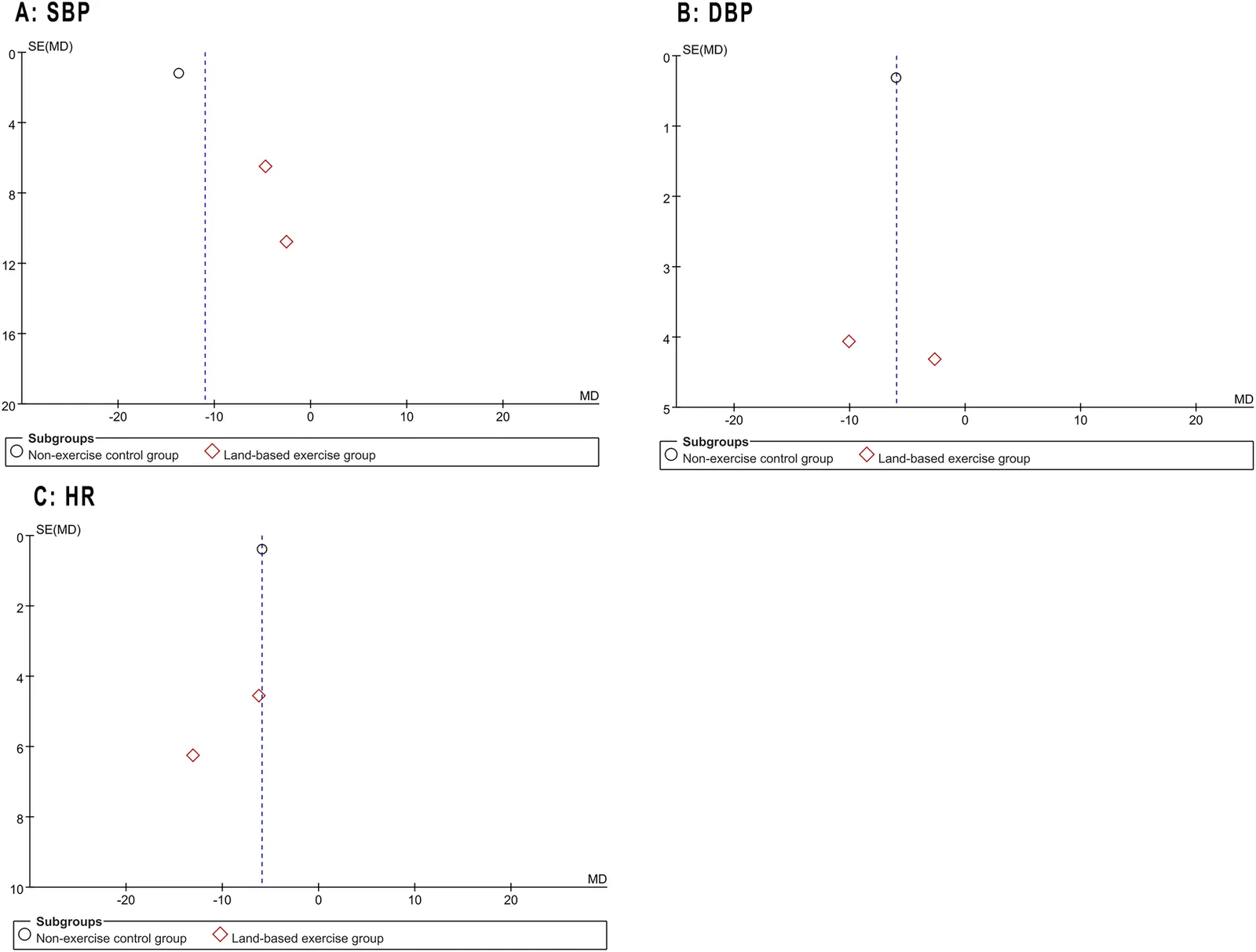
Funnel plot assessing publication bias for the primary outcomes.

### 3.9 Certainty of evidence

The certainty of the evidence for each outcome was assessed using the Grading of Recommendations Assessment, Development and Evaluation approach. The GRADE assessments are summarized in Table 5.

**Table 5:** Certainty of evidence assessment using the GRADE approach.

Certainty assessment							№ of patients		Effect		Certainty	Importance
№ of studies	Study design	Risk of bias	Inconsistency	Indirectness	Imprecision	Other considerations	[intervention]	[comparison]	Relative (95% CI)	Absolute (95% CI)		
SBP												
3	randomised trials	serious^a^	not serious	not serious	not serious	none	49	49	-	MD 10.98 lower (17.41 lower to 4.56 lower)	⊕⊕⊕◯ Moderate^a^	CRITICAL
DBP												
3	randomised trials	serious^a^	not serious	not serious	not serious	none	49	49	-	MD 5.97 lower (6.57 lower to 5.36 lower)	⊕⊕⊕◯ Moderate^a^	CRITICAL
HR												
3	randomised trials	serious^a^	not serious	not serious	not serious	none	49	49	-	MD 5.91 lower (6.7 lower to 5.12 lower)	⊕⊕⊕◯Moderate^a^	CRITICAL

CI: confidence interval; MD: mean difference.

a Some concerns or high risk of bias in included studies.

The certainty of evidence for systolic blood pressure (SBP), diastolic blood pressure (DBP), and heart rate (HR) was rated as moderate. The evidence for all three outcomes was downgraded by one level because of concerns regarding the risk of bias in the included studies. According to the RoB 2 assessment, some included studies were judged to have some concerns or a high risk of bias. No additional downgrading was applied for inconsistency, indirectness, imprecision, or publication bias.

Overall, the available evidence suggests that aquatic exercise therapy may improve blood pressure and heart rate in older adults with hypertension. However, the moderate certainty of evidence indicates that further well-designed randomized controlled trials are warranted to increase confidence in the current estimates and further clarify the effects of aquatic exercise therapy in this population.

## 4 Discussion

The present systematic review and meta-analysis synthesized evidence from four randomized controlled trials involving older adults with hypertension to evaluate the effects of aquatic exercise therapy on blood pressure and heart rate. The pooled results demonstrated that aquatic exercise therapy significantly reduced systolic blood pressure, diastolic blood pressure, and resting heart rate compared with the overall control interventions. Subgroup analyses according to comparator type showed significant reductions in SBP, DBP, and HR when AET was compared with non-exercise controls, whereas the pooled effects for SBP and DBP did not reach statistical significance when compared with land-based exercise. However, formal tests for subgroup differences did not demonstrate statistically significant differences between non-exercise and land-based exercise comparators for SBP (*P* = 0.09), DBP (*P* = 0.90), or HR (*P* = 0.47). These findings should therefore be interpreted cautiously, particularly given the small number of studies contributing to each comparator subgroup (5, 7, 10).

Although the overall findings support the beneficial effects of aquatic exercise therapy, considerable statistical heterogeneity was observed across several outcomes, and the certainty of evidence was rated as moderate according to the GRADE assessment. The observed heterogeneity may be attributable to differences in participant characteristics, including hypertension definitions, baseline blood pressure profiles, and antihypertensive medication status, as well as variations in intervention duration, exercise frequency, and comparator types among the included studies (29). In particular, the included RCTs differed in their eligibility criteria for hypertension and the reporting of medication use, with some studies including participants receiving regular antihypertensive treatment, whereas medication information was not available in all trials. Therefore, although the present findings support the potential role of AET as a lifestyle intervention for older adults with hypertension, they should be interpreted with appropriate caution, and further high-quality RCTs with standardized participant characteristics and intervention protocols are warranted to strengthen the evidence base.

The beneficial effects of aquatic exercise therapy observed in the present study may be explained by several physiological mechanisms unique to the aquatic environment. Unlike conventional land-based exercise, immersion in water exposes the body to buoyancy, hydrostatic pressure, viscosity, and thermoneutral water temperature, all of which produce distinct cardiovascular and musculoskeletal adaptations (10, 11). Among these factors, hydrostatic pressure is considered one of the principal mechanisms contributing to blood pressure reduction during aquatic exercise. Water immersion promotes the displacement of blood from the peripheral circulation to the thoracic cavity, thereby increasing venous return, cardiac preload, and stroke volume (11, 12). Consequently, cardiac output can be maintained with a lower heart rate, reducing myocardial oxygen demand and improving cardiovascular efficiency. Aquatic exercise may induce haemodynamic adaptations across different time frames. Acute responses following a single aquatic exercise session include transient increases in venous return due to hydrostatic pressure, enhanced peripheral circulation, and post-exercise reductions in blood pressure. With repeated training over several weeks, short-term adaptations may involve improvements in autonomic regulation, vascular function, and exercise tolerance, which may contribute to improved cardiovascular regulation. In the long term, repeated exposure to these physiological stimuli through regular AET may contribute to sustained reductions in resting BP and HR (10, 30). However, the magnitude of these adaptations may depend on variations in FITT parameters among studies, including differences in exercise intensity, frequency, duration, and aquatic exercise modality. For example, higher-intensity or longer-duration aquatic training may provide greater cardiovascular stimulus, whereas lower-intensity programs may be more suitable for older adults with limited exercise capacity. Therefore, the heterogeneity in intervention protocols should be considered when interpreting the pooled effects of AET.

In addition to its acute hemodynamic effects, aquatic exercise may improve vascular function through enhanced endothelial function and reduced arterial stiffness. Aerobic exercise increases laminar shear stress on the vascular endothelium, stimulating endothelial nitric oxide synthase activity and increasing nitric oxide bioavailability, which promotes vasodilation and decreases peripheral vascular resistance (31). Water immersion may further augment these responses by increasing central blood volume and vascular shear stress, thereby facilitating endothelial adaptation. Previous studies have also suggested that regular exercise can improve arterial compliance and reduce arterial stiffness, both of which are key determinants of systolic blood pressure in older adults (32). Given that age-related arterial stiffening is a major contributor to isolated systolic hypertension, the reduction in SBP observed in the present meta-analysis may be partly related to improved vascular regulation induced by regular aquatic exercise. Hydrostatic pressure enhanced venous return, and increased peripheral circulation during aquatic exercise may contribute to cardiovascular adaptations, including improved endothelial function and vascular responsiveness. However, because the included studies did not directly assess arterial stiffness or vascular function parameters, this potential mechanism remains hypothetical and requires confirmation in future studies.

Another potential mechanism involves modulation of autonomic nervous system activity. Warm-water immersion has been shown to suppress sympathetic nervous system activity while enhancing parasympathetic tone, resulting in reductions in resting heart rate and systemic vascular resistance. These autonomic adaptations may complement the cardiovascular effects of exercise training and contribute to improved blood pressure regulation. Furthermore, the buoyancy provided by water substantially reduces weight-bearing stress on the joints and lowers the risk of pain or instability during exercise. This allows older adults with obesity, osteoarthritis, impaired balance, or reduced physical function to perform aerobic exercise at an appropriate intensity with less discomfort than conventional land-based exercise (15). Consequently, aquatic exercise may provide an alternative exercise option for older adults who experience discomfort or difficulty during conventional land-based exercise. However, whether these characteristics translate into greater long-term exercise participation remains uncertain.

Despite these plausible mechanisms, the exact pathways through which aquatic exercise lowers blood pressure remain incompletely understood. None of the included studies directly assessed endothelial function, arterial stiffness, autonomic regulation, or neurohumoral responses, making it difficult to determine the relative contribution of each mechanism. Future randomized controlled trials incorporating objective measures of vascular function and autonomic activity are therefore needed to further elucidate the physiological mechanisms underlying the antihypertensive effects of aquatic exercise therapy.

The findings of the present review are generally consistent with previous evidence demonstrating that aquatic exercise is an effective non-pharmacological intervention for reducing blood pressure in individuals with hypertension. Previous systematic reviews have reported clinically meaningful reductions in both systolic and diastolic blood pressure following regular aquatic exercise, suggesting that water-based exercise provides cardiovascular benefits comparable to those of conventional aerobic exercise (33, 34). Although the magnitude of blood pressure reduction varies across studies, the overall direction of the treatment effect remains consistent with the present findings.

The comparator-specific analyses provide additional context for interpreting the effects of AET. When compared with non-exercise controls, AET was associated with significant reductions in SBP, DBP, and HR. However, the non-exercise control subgroup included only one study, which limits the certainty and generalizability of these findings. When compared with land-based exercise, AET was associated with a significant reduction in HR, whereas the pooled effects on SBP and DBP did not reach statistical significance. Importantly, the formal tests for subgroup differences did not demonstrate statistically significant differences between non-exercise and land-based exercise comparators for SBP (*P* = 0.09), DBP (*P* = 0.90), or HR (*P* = 0.47). Therefore, the differences observed in the statistical significance of individual subgroup estimates should not be interpreted as evidence that AET has different effects according to comparator type. The small number of available studies further limits the interpretation of these subgroup findings. Current international hypertension guidelines recommend aerobic exercise as an important lifestyle intervention because regular exercise can improve vascular endothelial function, reduce peripheral vascular resistance, and enhance autonomic cardiovascular regulation (35). Thus, AET may be considered an alternative mode of aerobic exercise for older adults with hypertension, particularly when participation in conventional land-based exercise is difficult. The buoyancy and reduced weight-bearing characteristics of water allow individuals with osteoarthritis, obesity, impaired balance, or reduced physical function to perform aerobic exercise with less discomfort and lower risk of falls. In older adults with hypertension, barriers to long-term participation in conventional land-based exercise may include reduced exercise tolerance, fatigue, concerns regarding exercise-induced cardiovascular responses or blood pressure fluctuations, musculoskeletal discomfort, and the coexistence of multiple chronic conditions. These factors may limit the initiation and maintenance of regular exercise participation. Consequently, although aquatic exercise may not produce greater blood pressure reductions than land-based exercise, its favourable safety profile and accessibility may provide an alternative exercise option for individuals who experience difficulties participating in conventional land-based programmes. However, the included studies did not directly assess exercise adherence; therefore, the potential effects of AET on long-term adherence require further investigation. These characteristics may be particularly relevant for older adults with hypertension, in whom sustained engagement in lifestyle interventions is important for cardiovascular risk management (6).

The present findings have several important clinical implications for the management of hypertension in older adults. Current international hypertension guidelines consistently recommend regular aerobic exercise as a cornerstone of non-pharmacological blood pressure management (5, 6). However, participation in conventional land-based exercise is often limited in older adults because of age-related conditions such as osteoarthritis, reduced muscle strength, impaired balance, obesity, and fear of falling. These barriers may reduce long-term adherence to exercise programmes and consequently diminish their antihypertensive benefits (36).

Aquatic exercise therapy may provide a practical and accessible alternative for this population. The buoyancy of water reduces weight-bearing stress on the lower extremities, allowing older adults to perform aerobic exercise with less joint pain and physical discomfort while maintaining an adequate training intensity. In addition, the supportive properties of water may increase confidence during movement and reduce the perceived risk of falling, thereby encouraging regular participation in physical activity. Although the present review did not identify statistically significant subgroup differences between aquatic exercise and land-based exercise for SBP or DBP, these findings should not be interpreted as evidence of equivalent effects. Nevertheless, aquatic exercise may provide an alternative exercise modality for older adults who experience difficulties participating in conventional land-based exercise. Its buoyancy and reduced weight-bearing characteristics may be particularly useful for individuals with musculoskeletal discomfort, impaired balance, or reduced physical function (15). However, because exercise adherence was not directly assessed in the included trials, whether aquatic exercise improves long-term participation remains uncertain.

The present systematic review and meta-analysis have several strengths. First, to our knowledge, this is the first systematic review and meta-analysis to specifically evaluate the effects of aquatic exercise therapy on blood pressure outcomes in older adults with hypertension, thereby providing focused evidence for a population at particularly high cardiovascular risk. Second, only randomized controlled trials were included, enhancing the overall methodological rigor of the evidence synthesis. Furthermore, subgroup analyses according to comparator type were conducted to explore potential sources of heterogeneity and to distinguish the effects of aquatic exercise from those of conventional land-based exercise. In addition, the certainty of evidence was systematically assessed using the GRADE approach, and publication bias was explored through funnel plot analysis, providing a more comprehensive evaluation of the robustness and reliability of the findings (22).

Several limitations should also be acknowledged. First, only four randomized controlled trials with relatively small sample sizes were included, which may have limited the statistical power of some pooled analyses and reduced the precision of the estimated treatment effects. Second, substantial heterogeneity was observed for several outcomes, which may be attributable to variations in participant characteristics, exercise protocols, intervention duration, training frequency, exercise intensity, and comparator interventions across studies (20). Third, although all participants included were older adults with hypertension, differences in baseline blood pressure levels, antihypertensive medication use, and comorbid conditions may have influenced the intervention effects. Fourth, the duration of follow-up was generally short, preventing an assessment of the long-term sustainability of the observed blood pressure reductions. Finally, although no obvious publication bias was identified based on visual inspection of the funnel plots, the small number of included studies limits the ability to reliably detect publication bias, as funnel plot asymmetry tests have limited power when few studies are available (22). Therefore, the findings should be interpreted with appropriate caution. Future large-scale, multicenter randomized controlled trials with standardized aquatic exercise protocols, longer follow-up periods, and comprehensive assessments of cardiovascular and functional outcomes are warranted to strengthen the current evidence base and further define the role of aquatic exercise therapy in hypertension management among older adults.

## 5 Conclusion

In conclusion, the present systematic review and meta-analysis suggests that aquatic exercise therapy (AET) may be an effective non-pharmacological intervention for improving blood pressure control in older adults with hypertension. Compared with control interventions, AET was associated with significant reductions in systolic blood pressure, diastolic blood pressure, and resting heart rate. Subgroup analyses according to comparator type showed that the magnitude of the estimated effects varied between non-exercise and land-based exercise comparators; however, these findings should be interpreted cautiously because of the limited number of studies within each subgroup. Although aquatic exercise may provide an alternative exercise option for older adults who experience difficulties participating in conventional land-based exercise, the included studies did not directly evaluate exercise adherence. Given the moderate certainty of evidence and the limited number and sample size of the included RCTs, the findings should be interpreted with caution. Future large-scale, high-quality RCTs with standardized intervention protocols and longer follow-up periods are warranted to confirm the long-term effectiveness of AET and to determine optimal exercise prescriptions for blood pressure management in older adults with hypertension.

## Data Availability

The original contributions presented in the study are included in the article/Supplementary Material, further inquiries can be directed to the corresponding author.
