# Supplementary material for: The effects of aquatic exercise therapy on blood pressure in elderly people with hypertension: a systematic review and meta-analysis
Source: Front Cardiovasc Med. 2026 Oct 8;13:1951070. doi: 10.3389/fcvm.2026.1951070 (PMC13687394; doi:10.3389/fcvm.2026.1951070)
Supplement: Supplementary file 1 [file Table1.docx]

| **P** | **I** | **C** | **O** | **S** |
| --- | --- | --- | --- | --- |
| Older adults with hypertension are diagnosed according to recognized clinical guidelines or physician diagnosis. Participants were generally aged ≥60 years or classified as older adults in the original studies, regardless of sex, ethnicity, disease duration, or antihypertensive medication use. | Structured aquatic exercise interventions performed in a water environment were eligible, including water-based aerobic exercise, aquatic resistance exercise, aquatic combined exercise, and aquatic exercise therapy. The included interventions were required to involve active, supervised, or prescribed physical exercise conducted in water. Swimming was considered eligible only when it was performed as a structured exercise intervention rather than recreational activity. | Conventional rehabilitation, land-based exercise, usual care, no intervention, or other control interventions | The primary outcomes were systolic blood pressure (SBP) and diastolic blood pressure (DBP). Secondary outcomes included mean arterial pressure (MAP), resting heart rate, cardiorespiratory fitness (e.g., VO₂peak, VO₂max, or the 6-Minute Walk Test), vascular function (e.g., flow-mediated dilation, pulse wave velocity, and arterial stiffness), body composition (e.g., body mass index, body weight, and waist circumference), health-related quality of life, and adverse events. | Randomized controlled trial (RCT) |

**Supplementary Table 1 The PICOs details**
