# Supplementary material for: The effects of aquatic exercise therapy on blood pressure in elderly people with hypertension: a systematic review and meta-analysis
Source: Front Cardiovasc Med. 2026 Oct 8;13:1951070. doi: 10.3389/fcvm.2026.1951070 (PMC13687394; doi:10.3389/fcvm.2026.1951070)
Supplement: Supplementary file 2 [file Table2.docx]

2026.7.17

PubMed: 73

Cochrane Library: 79

Embase: 182

CINAHL: 50

WEB OF SCIENCE: 380

PubMed:

( "Aquatic Therapy"[Mesh] OR aquatic therap*[Title/Abstract] OR aquatic exercise*[Title/Abstract] OR water-based exercise*[Title/Abstract] OR water exercise*[Title/Abstract] OR hydrotherap*[Title/Abstract] OR aquatic training[Title/Abstract] OR pool exercise*[Title/Abstract] OR aquatic rehabilitation[Title/Abstract] OR water-based rehabilitation[Title/Abstract] OR water walking[Title/Abstract] OR aqua jogging[Title/Abstract] OR aquatic resistance exercise[Title/Abstract] OR aquatic balance training[Title/Abstract] OR swimming[Title/Abstract] OR swim*[Title/Abstract] ) AND ( "Hypertension"[Mesh] OR hypertension[Title/Abstract] OR hypertens*[Title/Abstract] OR "high blood pressure"[Title/Abstract] ) AND ( aged[MeSH Terms] OR "Aged, 80 and over"[MeSH Terms] OR elderly[Title/Abstract] OR older adult*[Title/Abstract] OR older people[Title/Abstract] OR older person*[Title/Abstract] OR older patient*[Title/Abstract] OR aging[Title/Abstract] OR ageing[Title/Abstract] OR geriatric*[Title/Abstract] OR senior*[Title/Abstract] )

Cochrane Library:

[mh "Aquatic Therapy"] OR (aquatic therap*):ti,ab,kw OR (aquatic exercise*):ti,ab,kw OR (water-based exercise*):ti,ab,kw OR (water exercise*):ti,ab,kw OR (hydrotherap*):ti,ab,kw OR (aquatic training):ti,ab,kw OR (pool exercise*):ti,ab,kw OR (aquatic rehabilitation):ti,ab,kw OR (water-based rehabilitation):ti,ab,kw OR (water walking):ti,ab,kw OR (aqua jogging):ti,ab,kw OR (aquatic resistance exercise):ti,ab,kw OR (aquatic balance training):ti,ab,kw OR (swimming):ti,ab,kw OR (swim*):ti,ab,kw

And

[mh Hypertension] OR hypertension:ti,ab,kw OR hypertens*:ti,ab,kw OR "high blood pressure":ti,ab,kw

And

[mh Aged] OR [mh "Aged, 80 and over"] OR elderly:ti,ab,kw OR "older adult*":ti,ab,kw OR "older people":ti,ab,kw OR "older person*":ti,ab,kw OR "older patient*":ti,ab,kw OR aging:ti,ab,kw OR ageing:ti,ab,kw OR geriatric*:ti,ab,kw OR senior*:ti,ab,kw

Embase:

('aquatic therapy'/exp OR aquatic therap*:ti,ab,kw OR aquatic exercise*:ti,ab,kw OR water-based exercise*:ti,ab,kw OR water exercise*:ti,ab,kw OR hydrotherap*:ti,ab,kw OR aquatic training:ti,ab,kw OR pool exercise*:ti,ab,kw OR aquatic rehabilitation:ti,ab,kw OR water-based rehabilitation:ti,ab,kw OR water walking:ti,ab,kw OR aqua jogging:ti,ab,kw OR aquatic resistance exercise:ti,ab,kw OR aquatic balance training:ti,ab,kw OR swimming:ti,ab,kw OR swim*:ti,ab,kw) AND ('hypertension'/exp OR hypertension:ti,ab,kw OR hypertens*:ti,ab,kw OR 'high blood pressure':ti,ab,kw) AND ('aged'/exp OR 'very elderly'/exp OR elderly:ti,ab,kw OR 'older adult*':ti,ab,kw OR 'older people':ti,ab,kw OR 'older person*':ti,ab,kw OR 'older patient*':ti,ab,kw OR aging:ti,ab,kw OR ageing:ti,ab,kw OR geriatric*:ti,ab,kw OR senior*:ti,ab,kw)

CINAHL:

( MH "Aquatic Therapy+" OR TI (aquatic therap*) OR AB (aquatic therap*) OR TI (aquatic exercise*) OR AB (aquatic exercise*) OR TI (water-based exercise*) OR AB (water-based exercise*) OR TI (water exercise*) OR AB (water exercise*) OR TI (hydrotherap*) OR AB (hydrotherap*) OR TI (aquatic training) OR AB (aquatic training) OR TI (pool exercise*) OR AB (pool exercise*) OR TI (aquatic rehabilitation) OR AB (aquatic rehabilitation) OR TI (water-based rehabilitation) OR AB (water-based rehabilitation) OR TI (water walking) OR AB (water walking) OR TI (aqua jogging) OR AB (aqua jogging) OR TI (aquatic resistance exercise) OR AB (aquatic resistance exercise) OR TI (aquatic balance training) OR AB (aquatic balance training) OR TI (swimming) OR AB (swimming) OR TI (swim*) OR AB (swim*) ) AND ( MH "Hypertension+" OR TI hypertension OR AB hypertension OR TI hypertens* OR AB hypertens* OR TI "high blood pressure" OR AB "high blood pressure" ) AND ( MH "Aged+" OR TI elderly OR AB elderly OR TI "older adult*" OR AB "older adult*" OR TI "older people" OR AB "older people" OR TI "older person*" OR AB "older person*" OR TI "older patient*" OR AB "older patient*" OR TI aging OR AB aging OR TI ageing OR AB ageing OR TI geriatric* OR AB geriatric* OR TI senior* OR AB senior* )

WEB OF SCIENCE:

TS=(aquatic therap* OR aquatic exercise* OR water-based exercise* OR water exercise* OR hydrotherap* OR aquatic training OR pool exercise* OR aquatic rehabilitation OR water-based rehabilitation OR water walking OR aqua jogging OR aquatic resistance exercise OR aquatic balance training OR swimming OR swim*)

And

TS=(hypertension OR hypertens* OR "high blood pressure")

And

TS=(elderly OR "older adult*" OR "older people" OR "older person*" OR "older patient*" OR aging OR ageing OR geriatric* OR senior*)
