# Supplementary material for: The effects of aquatic exercise therapy on blood pressure in elderly people with hypertension: a systematic review and meta-analysis
Source: Front Cardiovasc Med. 2026 Oct 8;13:1951070. doi: 10.3389/fcvm.2026.1951070 (PMC13687394; doi:10.3389/fcvm.2026.1951070)
Supplement: Supplementary file 3 [file Table3.docx]

**Supplementary Table 3 Risk of bias**

| Study ID | Randomization process | Deviations from intended interventions | Missing outcome data | Measurement of the outcome | Selection of the reported result | Overall Bias |
| --- | --- | --- | --- | --- | --- | --- |
| Júnior et al (2020, English, Brazil) | Some concerns | Some concerns | Some concerns | Low | Some concerns | Some concerns |
| Kim et al (2016, English, Korea) | Some concerns | Low | Some concerns | Low | Some concerns | Some concerns |
| Ruangthai et al (2020, English, Thailand) | Some concerns | Some concerns | Some concerns | Low | Some concerns | Some concerns |
| Sosner et al (2019, English, Canada) | Some concerns | Low | Some concerns | Low | Some concerns | Some concerns |
