# Supplementary material for: The effects of aquatic exercise therapy on blood pressure in elderly people with hypertension: a systematic review and meta-analysis
Source: Front Cardiovasc Med. 2026 Oct 8;13:1951070. doi: 10.3389/fcvm.2026.1951070 (PMC13687394; doi:10.3389/fcvm.2026.1951070)
Supplement: Supplementary file 4 [file Table4.docx]

**Supplementary Table 4 Overall effects**

| **Outcome** | **95% CI, I2** | **P value** |
| --- | --- | --- |
| SBP | -10.98 [-17.41 to -4.56], I² = 29% | 0.001** |
| DBP | -5.97[-6.57 to -5.36], I² = 0% | 0.001** |
| HR | -5.91[-6.7 to -5.12], I² = 0% | 0.001** |

Note: SBP, systolic blood pressure; DBP, diastolic blood pressure; HR, heart rate.

Difference *: p ＜ 0.05; **: p ＜ 0.01
