# Supplementary material for: The effects of aquatic exercise therapy on blood pressure in elderly people with hypertension: a systematic review and meta-analysis
Source: Front Cardiovasc Med. 2026 Oct 8;13:1951070. doi: 10.3389/fcvm.2026.1951070 (PMC13687394; doi:10.3389/fcvm.2026.1951070)
Supplement: Supplementary file 5 [file Table5.docx]

**Supplementary Table 5 Subgroup analysis according to comparator type**

| **Outcome** | **Subgroup** | **No. of studies** | **95% CI, I2** | **P value** | **Test for subgroup differences** |
| --- | --- | --- | --- | --- | --- |
| SBP | Non-exercise control group | 1 | -13.64 [-16.05, -11.23], I² = Not applicable | 0.001** | χ²=2.80, df=1, P=0.09, I²=64.3% |
|  | Land-based exercise group | 2 | -4.11 [-15.01,6.78], I² = 0% | 0.46 |  |
| DBP | Non-exercise control group | 1 | -5.96 [-6.57, -5.35], I² = Not applicable | 0.001** | χ²=0.02, df=1, P=0.90, I²=0% |
|  | Land-based exercise group | 2 | -6.44 [-13.69, 0.81], I² = 36% | 0.21 |  |
| HR | Non-exercise control group | 1 | -5.88 [-6.67, -5.09], I² = Not applicable | 0.001** | χ²=0.53, df=1, P=0.47, I²=0% |
|  | Land-based exercise group | 2 | -8.56 [-15.76, -1.36], I² = 0% | 0.02* |  |

Note: SBP, systolic blood pressure; DBP, diastolic blood pressure; HR, heart rate.

Difference *: p ＜ 0.05; **: p ＜ 0.01
